# A new grid- and modularity-based layout algorithm for complex biological networks

**DOI:** 10.1371/journal.pone.0221620

**Published:** 2019-08-29

**Authors:** Sheng He, Yi-Jun Liu, Fei-Yue Ye, Ren-Pu Li, Ren-Jun Dai

**Affiliations:** School of Computer Engineering, Jiangsu University of Technology, Changzhou, China; Beijing University of Posts and Telecommunications, CHINA

## Abstract

The visualization of biological networks is critically important to aid researchers in understanding complex biological systems and arouses interest in designing efficient layout algorithms to draw biological networks according to their topology structures, especially for those networks with potential modules. The algorithms of grid layout series have an advantage in generating compact layouts with overlap-free nodes compared to force-directed; however, extant grid layout algorithms have difficulty in drawing modular networks and often generate layouts of high visual complexity when applied to networks with dense or clustered connectivity structure. To specifically assist the study of modular networks, we propose a grid- and modularity-based layout algorithm (GML) that consists of three stages: network preprocessing, module layout and grid optimization. The algorithm can draw complex biological networks with or without predefined modules based on the grid layout algorithm. It also outperforms other existing grid-based algorithms in the measurement of computation performance, ratio of edge-edge/node-edge crossings, relative edge lengths, and connectivity F-measures. GML helps users to gain insight into the network global characteristics through module layout, as well as to discern network details with grid optimization. GML has been developed as a VisANT plugin (https://hscz.github.io/Biological-Network-Visualization/) and is freely available to the research community.

## Introduction

Network diagrams provide a fundamental conceptual framework for visualizing and mining high-throughput biological datasets, as well as for gaining insights and interpreting the biological implications by means of graph drawing algorithms [1–3]. With the advances in biotechnology, biological datasets have rapidly increased in size and complexity, bringing more challenges to network-based data visualization[4–5]. For complex biological networks composed of thousands of nodes, drawing algorithms may strive to grasp the global characteristics of networks to clarify their complexity [2,6–7].

Modularization is one of the most significant global characteristics of biological networks, where closely connected nodes (e.g., biomolecules) are usually organized as a module to carry out a specific function [4,8–9]. Biological modules can be generated through clustering algorithms (i.e., pseudomodules) that aim to identify sets of closely connected nodes from networks. Such modular architecture is significantly beneficial to reducing network complexity and helping researchers understand inherent biological implications when complex networks are divided into pseudo- or predefined modules [10–11].

A biological module has two different states, expanded and collapsed. Expanded modules display an internal subgraph (that is, places all descendent nodes with their connections into the graph), while collapsed modules replace this subgraph with a single node [10]. This results in problems with multiscale visualization when integrating information at different abstraction scales into one network and makes drawing the network much more difficult because both the modules and many descendant nodes embedded in these modules need to be handled simultaneously.

Network layout algorithms seek to place nodes in 2-dimensional (2D) space according to the network topology. The force-directed classical algorithm, which is available in most visualization tools (e.g., VisANT [12], Cytoscape [13–14] and CellDesigner [15]), models a network as a physical system with repulsive forces assigned among all nodes and attractive forces assigned between adjacent nodes [16]. In the layout of force-directed series, the adjacent nodes are inclined to locate too closely and easily overlap each other [17–18]. To address the problem, a grid layout algorithm has been proposed to place the nodes on grid points to avoid node overlap, and the interactions among nodes are specified with a cost function that is designed based on the topological structure of the network. Grid layout algorithms generate compact and biologically comprehensible layouts at the expense of computation performance [18–20]. To decrease computation costs, some algorithms are used in rigorous studies to improve the grid-optimization process through special methods (e.g., *reoptimization-after-perturbation* [18], *sweep calculation* [19] and *subcellular localizations* [21]). These algorithms outperform for small-sized networks of several hundred nodes, whereas larger networks, especially those with a dense connectivity structure, are hard to use to yield informative drawings, often leading to *hairball* layouts [2].

Recently, implicit modular information has been utilized to enhance the performance of grid layout algorithms for the visualization of complex networks. Inoue et al. [20] presented a hybrid grid layout algorithm using four different approaches, i.e., Spectral Analysis (H-SA), Kamada-Kawai (H-KK), Fruchterman-Reingold (H-FR) and Gursoy-Atun (H-GA), to preprocess the networks before distributing nodes to grid points. In particular, Spectral Analysis is a clustering algorithm to divide complex networks into modules. He *et al*. [22] designed a grid-based layout algorithm (GBL) that is specifically focused on drawing complex networks with modular properties, where all modules are placed randomly on separated 2D spaces (fan-shaped area). Compared with previous grid-series algorithms, both module-based algorithms show better performance in network visualization characteristics such as computation speed, edge-edge crossings, node-edge crossings, relative edge length and connectivity F-measures; however, the connectivities among modules of output graphs remain poorly understood, and these graphs still appear with high visual complexity, in that the modules are not organized deliberately or located properly in a hierarchical structure.

**Fig 1:**
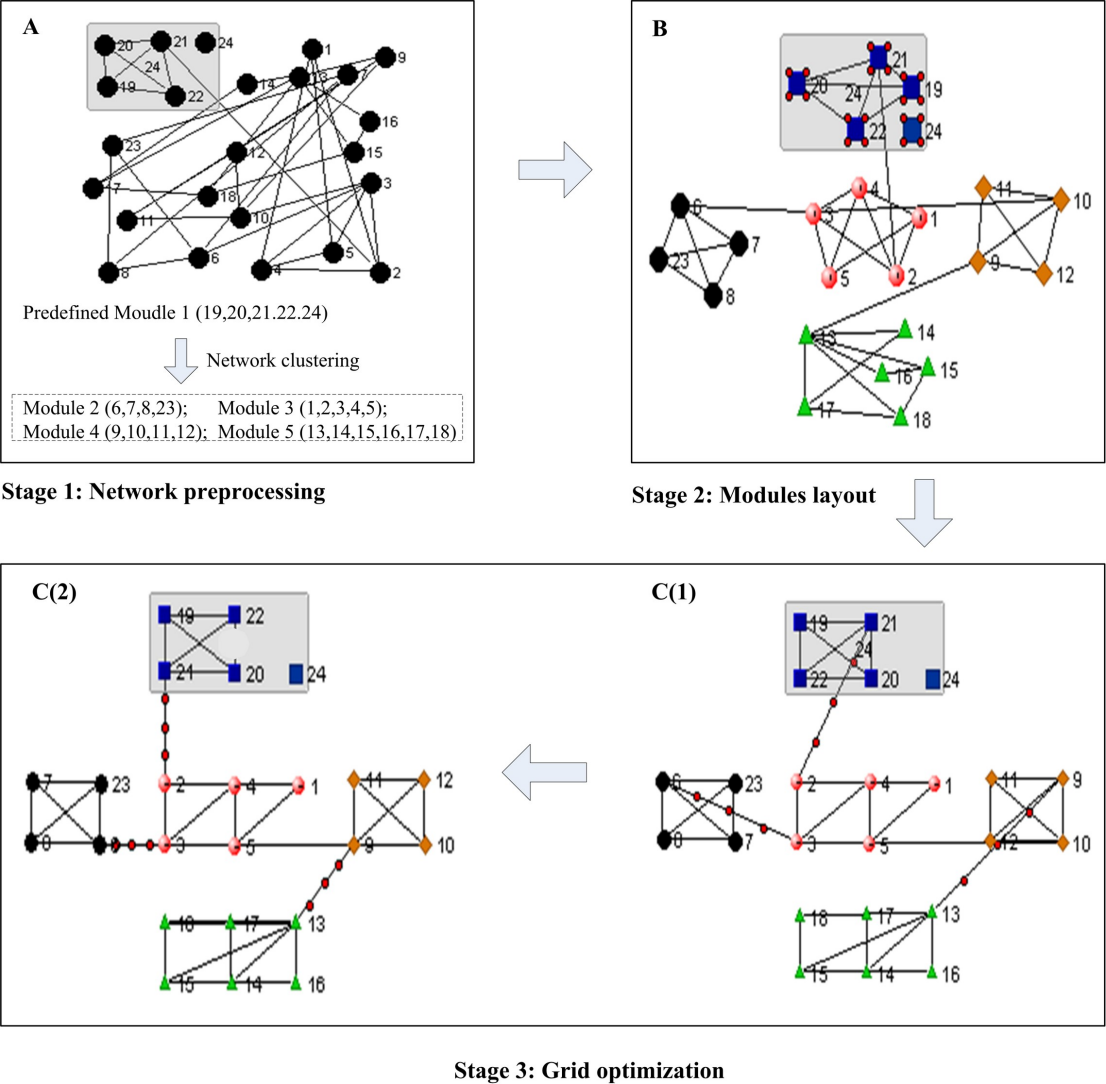
The flow chart of the GML algorithm. A network of 24 nodes is used for illustration of GML procedures. A) Nodes within the predefined modules (node 19, 20, 21, 22 and 24) are filtered out and the remainder is partitioned into modules using a clustering algorithm. Node 24 in the predefined module has no connection to the rest of nodes. B) All modules are placed on the plane according to the method of module layout, where each module is expanded to the square area to ensure a large enough space for placements of the nodes embedded in the module. C) The positions of all nodes are optimized in accordance with their inter/intramodule connectivity simultaneously to minimize edge-edge crossings through the method of grid optimization.

We propose a new algorithm named the grid- and modularity-based layout (GML) that is specifically focused on the modular properties of complex biological networks, where both predefined and pseudo modules are arranged and placed on a 2D plane. GML optimizes the node positions according to their modularity and connectivity through three stages (see Fig 1). First, the nodes of some acquired metabolic pathways or protein complexes with special functions are grouped as predefined modules, and then a clustering algorithm is applied to divide the remaining nodes into pseudo modules in the network preprocessing stage; a module-layout method is then executed to position all modules (regarded as single nodes) based on their intermodule connectivities; and finally, the method of grid optimization is applied to position the descendant nodes of the modules based on the inter- and intra-module connectivities.

## Methods

A biological network can generally be converted into a graph where molecules are represented as geometric points (nodes) and interactions between molecules are represented as straight lines (edges). Under such a drawing convention, a layout can be achieved when all node coordinates are determined through a specific optimization procedure to fulfill certain criteria. Given a network of *n* nodes, the layout **R** can be denoted by **R** = **R**(**r**_1_, **r**_2_,…, **r**_*n*_) where **r**_*i*_ = (*x*_*i*_, *y*_*i*_) represents the coordinates. In the grid layout algorithm, all *x*_*i*_ and *y*_*i*_ are integers because the nodes are restricted to grid points [17–18].

### The original grid layout

#### The cost function of original grid layout

The original grid layout aims to find the best layout by optimizing a cost function *C* **(W, R)**[17]:

$$C(W,R)=\sum \;w_{i\;j}*d_{i\;j}$$ (1)

where *w*_*i j*_ is the weight between nodes *i* and *j*, and the weights between all node pairs constitute the weight matrix **W**. **W** represents the topology structure of the network and is set according to the path lengths, which are defined as the number of steps along the shortest paths of node-pairs. For detailed explanations of path length, please refer to [23]. The term *d*_*i j*_ is the Manhattan distance between nodes *i* and *j*. In general, the weights between closely related nodes are assigned larger values to place node-pairs close.

#### The procedures of original grid layout

An optimization procedure termed *reoptimization-after-perturbation* is used in original grid optimization to improve algorithm performance [18]. The procedure starts with a random layout, **R,** which undergoes the partial optimization that moves every single node to its adjacent vacant grid point to reduce the cost *C* **(W, R)**. To avoid the local minimum and achieve the full optimization of **R**, the layout is perturbed by moving each node to a randomly chosen neighboring grid point with a given probability. The perturbed layout **R’** is once again optimized, and the layout of the smaller cost score is selected as the new input to repeat the optimization procedure until it reaches sufficient iteration time.

### Grid- and Modularity-based layout (GML)

#### The cost function of GML

When the network includes modules, the optimization procedure proceeds in two phases:

- Module layout: the positions of modules (represented by the points) are optimized and all modules are expanded and placed on the 2D plane.
- Grid optimization: the positions of all descendent nodes of the modules are optimized, and they are placed at grid points with minimized edge-edge crossings.

Modified from the original grid layout, the cost functions of optimizations are denoted as follows:

$$C_{module}(W_{module},R_{module})=\sum w_{h\;k}*d_{h\;k}\;Module\;layout$$ (2)

$$C_{node}(W_{node},R_{node})=\sum w_{i\;j}*d_{i\;j}\;Grid\;optimization$$ (3)

- *w*_*h k*_: Weight of module *h* and *k*, the weights between all module-pairs constitute the weight matrix **W**_*module*._
- *d*_*h k*_: Manhattan distance between module *h* and *k* in the module layout.
- *w*_*i j*_: Weight of descendent node *i* and *j*; the weights between all node-pairs constitute the weight matrix **W**_*node*._
- *d*_*i j*_: Manhattan distance between descendent nodes *i* and *j* in the layout of the stage of grid optimization.

The variable *d*_*h k*_ is defined by Eq (4) where (*x*, *y*) represents the coordinates of module and larger *d*_*h k*_ means greater physical distance between modules *h* and *k* (represented by the points). It should be noted that *d*_*h k*_ is defined as Manhattan distance rather than Euclidean distance because the former has the advantage of less computation.

$$d_{h\;k}=|x_{h}-x_{k}|+|y_{h}-y_{k}|$$ (4)

The network layout of GML is evaluated by two cost functions (*C*_*module*_ for the phase of module layout and *C*_*node*_ for grid optimization), which are defined as Eqs (2) and (3), respectively.

#### Procedures of the GML algorithm

As shown in Fig 1, GML draws complex biological networks in three stages.

**Stage 1**. Network preprocessing

The nodes of a biological network can be partitioned into modules using clustering algorithms, except those embedded in the predefined modules, which have specific biological functions and are predefined by researchers. The nodes within the same modules usually have dense internal connections but sparse connections to the rest of the network, and the resulting modules are often enriched with certain biological functions [24–25]. Note that predefined modules may or may not have such topological features because they can be defined by varied biological backgrounds, as shown in Fig 1A. The multilevel clustering algorithm has relatively high network modularity and low computation time compared to conventional single-level algorithms; therefore, it has been selected as the network preprocessing algorithm in this study. The computational complexity of multilevel clustering is ***O* (*m* log *n*)**, where *m* and *n* are the number of edges and nodes of the biological network, respectively [26].

**Stage 2**. Modules layout

**Fig 2:**
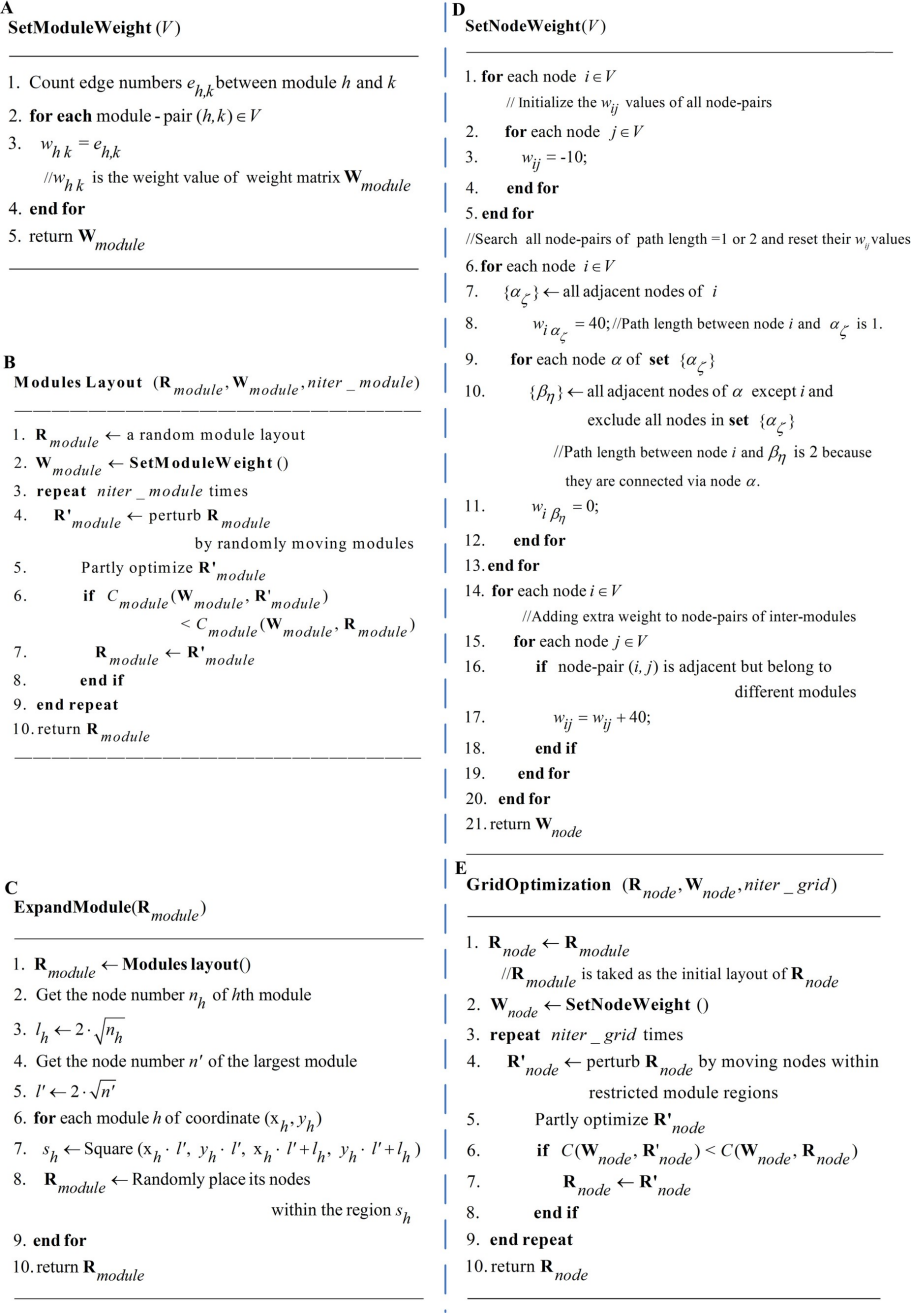
The pseudocodes of GML. The 2^nd^ stage of GML (Stage 2) includes the child processes (SetModuleWeight, ModuleLayout and ExpandModule) which are described by pseudocode A, B and C, and the 3^rd^ stage of GML (Stage 3) includes child processes (SetNodeWeight and GridOptimization) that are described by pseudocode D and E.

As shown in Fig 1B, the focus of this stage is to place all modules in a 2D plane. There are two tasks in this stage: 1) determination of the relative positions of the modules; and 2) determination of the size of each module. For the first task, a new weight-setting strategy for module-pairs is designed to improve algorithm performance, and the cost function *C*_*module*_ in Eq (2) is optimized to determine the module positions. As shown in Fig 2A, for any module *h* and *k*, the weight value *w*_*h k*_ of **W**_*module*_ is set according to the number of edges (represented by variable *e*_*h k*_) between the nodes embedded in modules *h* and *k*. The module-pairs with many more edges are assigned larger weight values to force them to be located close together, therefore reducing the edge-edge crossings between modules.

For a network of *n* nodes and *m* edges, the computational complexity can be estimated as ***O* (*n* ·*m*)** in the worst-case because the number of modules is not larger than *n*.

The procedure of module layout is shown in Fig 2B. All modules are regarded as single points and placed on grid points of a 2D plane. Then, their positions are optimized based on the weight matrix **W**_*module*_ and iteration parameter *niter_module* through the *reoptimization-after-perturbation* strategy.

The second task involves two main considerations. First, each module needs enough space not only to host its descendant nodes but also to clearly display the connections between these nodes. Second, the modules shall not overlap each other. From this perspective, the size of each modules is estimated by the function ExpandModule (**R**_*module*_) and the pseudocode, shown in Fig 2C.

Each module is bound by a square area whose side length *l*_*h*_ is empirically estimated based on 2·√*n*_*h*_ where *n*_*h*_ is the number of nodes embedded in the *h*^th^ module. The term Square (*x*_*h*_ · *l’*, *y*_*h*_ · *l’*, *x*_*h*_ · *l’*+ *l*_*h*_, *y*_*h*_ ·*l’*+ *l*_*h*_*)* in Fig 2C represents the square region with edge length *l*_*h*_ for the module *h* whose upper-left coordinate is (*x*_*h*_·*l’*, *y*_*h*_ ·*l’)*. The multiplication of the coordinate (*x*_*h*_, *y*_*h*_) for module *h* with edge length *l* ‘ (estimated based on the largest module) is designed to ensure enough distance between adjacent modules to avoid potential overlaps, as shown in Fig 1B. The stage of module layout takes no more than ***O* (*n***^**2**^**)** time in the worst case because the number of modules and their descendant nodes are both less than *n*.

**Stage 3**. Grid optimization

The key challenge in this stage is to optimize the positions of all descendant nodes embedded in the modules based on their inter- and intramodule connectivities simultaneously with minimized edge-edge crossings, as detailed in Fig 1C. First, for the descendant nodes embedded in the same module (node-pairs of intramodules), the weight values are set according to the path lengths between node-pairs through searching their adjacent nodes. Following the criteria of the algorithms of the grid layout series, the node-pairs of smaller path lengths are assigned higher weights and those of larger path lengths are assigned lower or negative weights [17]. The weight values for the node-pairs with path length 1, 2, 3 and beyond have been set as 40, 0 and -10, respectively. For a given network *V*, the procedures of searching path lengths and generating the weight matrix are shown in the function SetNodeWeight (*V*) in Fig 2D (line 1–13). Note that the weight values (i.e., 40, 0, -10) are assigned empirically based on comprehensive tests. Second, for the nodes belonging to different modules and path length = 1 (node-pairs of intermodules), the extra weight values are added so that their positions will be automatically oriented to the corresponding modules to minimize edge-edge crossings. As shown in Fig 1C(1), there are serious edge-edge crossings due to the improper position of node pairs (2, 21), (9, 13), and (3, 6), which can be eased if the locations of node 6, 9 and 21 are optimized as shown in Fig 1C(2). We apply a direct and efficient strategy to address this problem by adding an extra weight value of node-pairs between modules so that they will be attracted to the right sides of the corresponding modules. The detailed procedure of weight matrix adjustment is also shown in Fig 2D (line 14–20), where the extra weight is set as an empirical value to 40. Finally, the positions of all nodes are optimized based on the weight matrix **W**_*node*_ (generated by SetNodeWeight(*V*)), and the iteration parameter *niter_grid* through *reoptimization-after-perturbation* strategy and the pseudocodes of grid optimization are shown in Fig 2E.

The time cost of the grid optimization stage is focused on function SetNodeWeight (*V*) whose time complexity can be estimated as ***O*** (***n***^2^). The time cost of the initialization process (lines 1–5 in Fig 2D) is ***O*** (***n***^2^). Lines 6–13 in Fig 2D show the procedure to search for adjacent nodes where the inner loop (lines 9–12) is designed to search the adjacent nodes of path = 2. Because the time cost for the inner loop is no more than ***O*** (***n***), the cost time for the dual loop is, therefore, also ***O*** (***n***^2^). The weight adjustments (lines 14–20) between intermodules take no more than ***O* (*n***^***2***^**)** time in the worst case.

## Implementation

**Fig 3:**
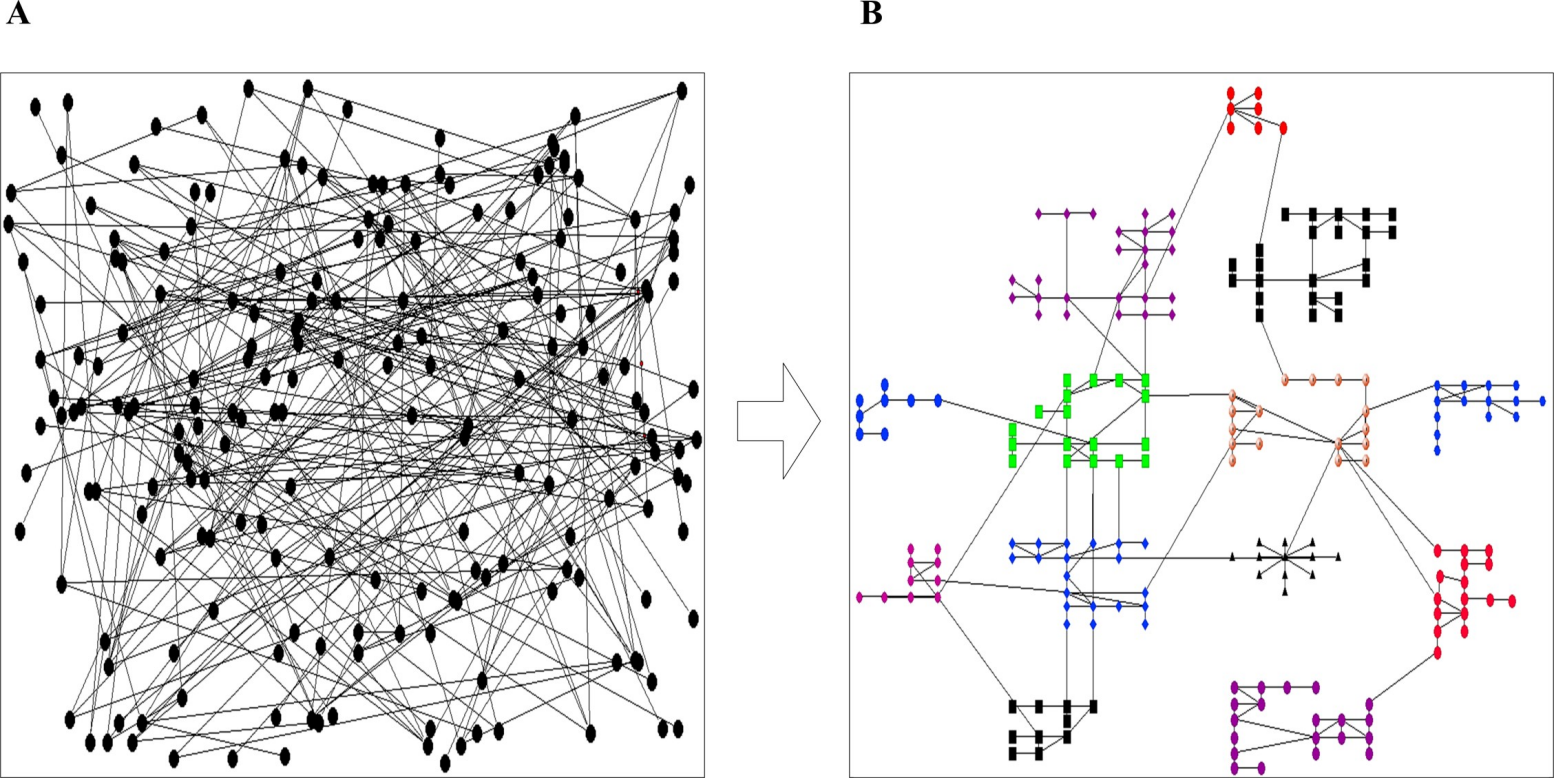
A layout of the yeast cell cycle regulatory network of 200 nodes visualized by the GML algorithm. A) random layout: the nodes were positioned randomly; B) GML layout: the nodes were optimized with the GML algorithm.

GML has been implemented as a VisANT plugin and developed in Java. As an integrative platform for the visual analysis of biological networks, VisANT (http://www.visantnet.org) provides rich graphical functionalities to support interactive operations on the graph drawings and manipulations. It also provides the simple but flexible plugin API (Application Interface) to make it easier to quickly develop the plugins. Fig 3 is an example network drawn using the GML algorithm. By means of clustering algorithms, the network is divided into 13 modules that are marked with different shapes and colors. The positions of modules and their descendant nodes are optimized through module-layout and grid-optimization, respectively, which separates the modules clearly, while their descendant nodes are placed relatively even in the output layout.

## Results

### Comparison with other grid layout algorithms

There are six primary extant grid layout algorithms, including the original grid layout [17], LucidDraw [18], hybrid grid layout [20], Cerebral [21], GBL layout [22] and SCCB-grid layout [27]. The hybrid grid layout and GBL are modularity -based algorithms that achieve relatively good performance compared with other grid layouts [20,22]. From this perspective, they have been chosen as the reference algorithms to be compared against GML. The performance of the hybrid grid layout is enhanced through four different preprocessor algorithms: H-SA, H-KK, H-FR, and H-GA. In the following comparison sections, all selected algorithms are compared independently with their default parameter settings.

Ten example networks have been selected to evaluate the performances for comparison purposes (Table 1). These networks involve three different types (regulatory network, protein-protein interaction network, and metabolic network) that represent a relatively wide range of topological properties.

**Table 1:** The list of example networks used to evaluate the performance of selected algorithms.

Name	Type	Nodes number	Edges number	Density	Mean node degree	Ref.
1. Yeast cell cycle	regulatory network	200	270	0.0129	2.580	[28]
2. Utez-screen	protein-protein interaction	263	292	0.0082	2.179	[29]
3. Subnetwork of PAO1	metabolic network	290	374	0.0089	2.581	[30]
4. Ito-core	protein-protein interaction	426	568	0.0062	2.556	[31]
5. Y2H-CCSB	protein-protein interaction	964	1598	0.0032	3.200	[32]
6. PAO1	metabolic network	1294	1590	0.0019	2.449	[30]
7. *L*. *lactis*	metabolic network	1489	3172	0.0029	4.260	[33]
8. *S*.*cerevisiae* iFF708	metabolic network	2879	5616	0.0013	3.884	[34]
9. *Aspergillus niger*	metabolic network	3774	7967	0.0012	4.229	[35]
10. *Aspergillus oryzae*	metabolic network	4976	11042	0.0009	4.446	[36]

All evaluations are based on averages of 10 runs on a Dell laptop (OS: windows 7 64 bit, CPU: Intel Core i3-2330M 2.20 GHz, memory: 8.00 GB). All experimental data can be found in S1 Table.

#### Computational efficiency

As discussed in the Methods section, the time complexity of every stage is listed in Table 2, and the time complexity of GML can be estimated as ***O* (*m* log *n*)** + ***O* (*m n*)**+ ***O* (*n***^***2***^**)** + ***O* (*n***^***2***^**)** = ***O* (*n* (*m + n*))** for a network of *m* edges and *n* nodes.

**Table 2:** Time complexity of GML (for a network of *n* nodes and *m* edges).

Three procedures of GML	Time complexity	Description
Stage 1: Network preprocessing	***O* (*m* log *n*)**	Using clustering algorithm (multilevel clustering) to divide the networks into module;
Stage 2: Modules layout	***O* (*n* ·*m*)**	Determining the relative positions of the modules;
	***O* (*n***^**2**^**)**	Determining the size of each module;
Stage 3: Grid optimization	***O* (*n***^**2**^**)**	Optimizing the positions of all descendant nodes embedded in the modules

**Fig 4:**
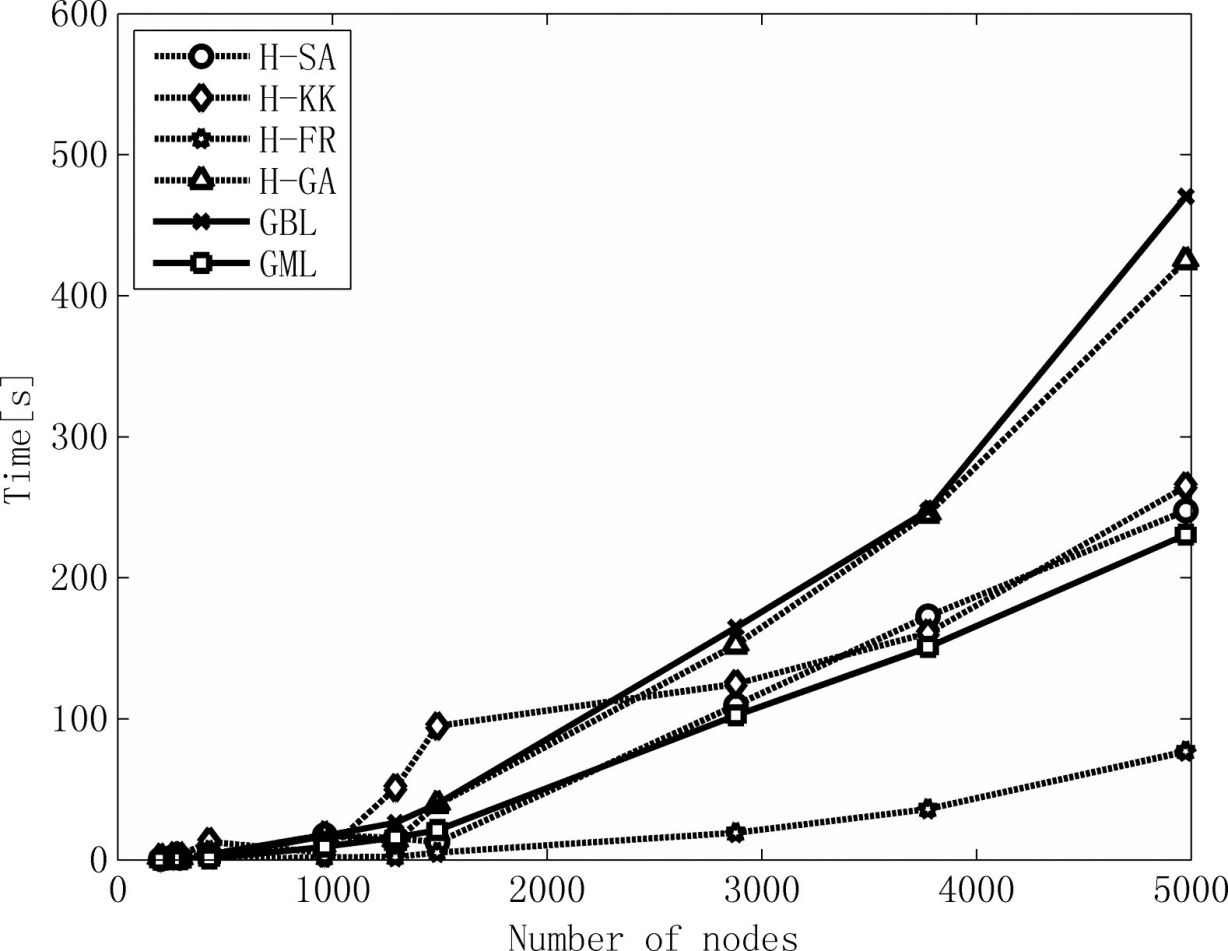
The comparison of computation time. The computation time for each test network was averaged on 10 runs on a Dell laptop with an Intel Core i3-2330M processor.

We evaluated the computational efficiency of GML by comparing its computation time against those of the hybrid grid layout and GBL, as shown in Fig 4. In general, H-FR is the fastest algorithm while GBL is the slowest one, and GML outperforms the other four layout algorithms, except H-FR.

#### Modular characteristics

Two measurements characterize the modular features of the GML algorithm.

**Connectivity F-measure.** The connectivity F-measure evaluates whether nodes with dense connectivities are positioned together [20,37]. It is defined as the weighted harmonic average of precision and recall, which are widely used in the area of information retrieval [37].

On a 2D plane, let #*S* be the number of nodes in set *S* and node *i* be the geometric center of circle *B*_*i*_(*r*_*i*_), the precision *P*_*i*_(*r*_*i*_) for the *i-*th *B*_*i*_(*r*_*i*_) is defined as

$$P_{i}(r_{i})=\frac{\#\{j|s_{j}\in B_{i}(r_{i}),a_{i\;j}=1,j\neq i\}}{\#\{j|s_{j}\in B_{i}(r_{i}),j\neq i\}},$$ (5)

where *s*_*j*_ is node *j* within circle *B*_*i*_ and *r*_*i*_ is the radius of circle *B*_*i*_. If node *j* is adjacent to node *i*, the term *a*_*i j*_ = 1. The precision *P*_*i*_(*r*_*i*_) is the ratio of all the adjacent ones of node *i* to all nodes located in *B*_*i*._

Next, the recall *R*_*i*_(*r*_*i*_) is defined as:

$$R_{i}(r_{i})=\frac{\#\{j|s_{j}\in B_{i}(r_{i}),a_{i\;j}=1,j\neq i\}}{\#\{j|a_{i\;j}=1,j\neq i\}},$$ (6)

**Fig 5:**
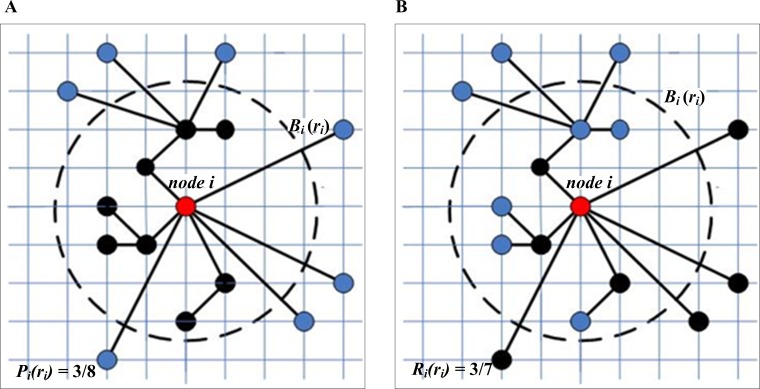
The examples of *P*_*i*_(*r*_*i*_) and *R*_*i*_(*r*_*i*_). A) Within the circle *B*_*i*_(*r*_*i*_), node *i* has three adjacent nodes out of the eight nodes (black), and the precision value *P*_*i*_(*r*_*i*_) is 3/8; B) Node *i* has seven adjacent nodes (black) on the 2D plane and three adjacent nodes within the circle *B*_*i*_(*r*_*i*_); therefore, the recall value *R*_*i*_(*r*_*i*_) is 3/7.

*R*_*i*_(*r*_*i*_) is the ratio of all the adjacent nodes of node *i* to all adjacent nodes in the network wherever they are located in *B*_*i*_. For a detailed explanation of *P*_*i*_(*r*_*i*_) and *R*_*i*_(*r*_*i*_), please see the examples in Fig 5.

Generally, higher precision favors a smaller *r*_*i*_, and higher recall favors a larger *r*_*i*_; the optimal radius *r*_*i*_ should be found in between. The optimal radius *r*_*i*_ for each *i* is selected that maximizes the following *F*-measure with weight α [20,37], as shown in Eq (7) (α = 0.5 is used throughout our experiments).

$$F_{i}(r_{i})=1/\left\{\alpha \frac{1}{P_{i}(r_{i})}+(1-\alpha )\frac{1}{R_{i}(r_{i})}\right\}.$$ (7)

Finally, in the 2D plane, the connectivity F-measure is defined as the average over all *N* nodes:

$$F=\sum_{i=1}^{N}\frac{F_{i}(r_{i})}{N}.$$ (8)

From Eq (7), we can learn that larger *P*_*i*_(*r*_*i*_) and *R*_*i*_(*r*_*i*_) values will generate larger *F*_*i*_(*r*_*i*_), which means that more adjacent nodes are located within *B*_*i*_(*r*_*i*_). Therefore, *F* can evaluate whether densely connected nodes are placed together in layouts.

**Relative edge length.** The relative edge length is used to examine whether the distributions of nodes are compact in the layout space and is defined as the ratio of the sum of all edge lengths (the Manhattan distance between connected nodes) divided by the product of the area of layout space and the number of total edges. Larger connectivity F-measure and shorter relative edge length indicate better modular characteristics [22].

#### Visual complexity

The ratio of edge-edge crossings and the ratio of node-edge crossings are used to assess the visual complexity of GML layouts. The former is defined as the number of edge-edge crossings divided by the total number of edge pairs, and the minimization of the ratio will avoid high visual complexity; the latter is defined as the number of node-edge crossings divided by the total number of node-edge pairs [19,27,38–39], and decreasing this ratio will avoid the misunderstanding of network connectivity when edges cross the nodes. In short, a smaller ratio of edge-edge crossings and node-edge crossings means better layouts.

**Fig 6:**
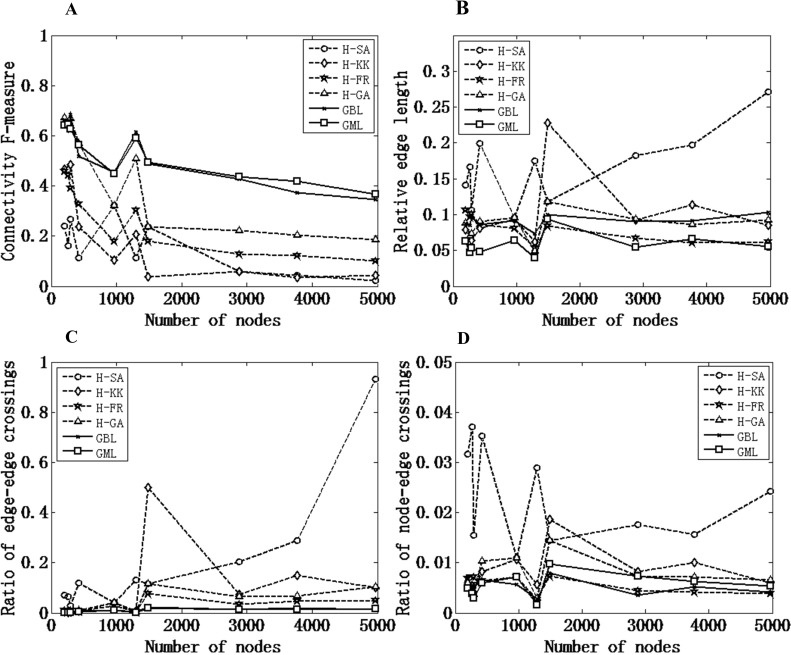
Characterization of the GML algorithm. The hybrid layout algorithms with different preprocessors (H-SA, H-KK, H-FR, and H-GA) and GBL are used as reference methods for comparison purposes. A) connectivity F-measure, B) relative edge length, C) ratio of edge-edge crossings, and D) ratio of node-edge crossings.

Fig 6 shows the characterization of the GML algorithm. To grasp the key features of the algorithms, all measurements are summarized in Table 3, where each algorithm is ranked according to Figs 4 and 6. For example, if H-FR performs the best and GBL the worst in computational efficiency among six selected algorithms, then the rank value of H-FR is 1 and that of GBL is 6.

**Table 3:** Characterizations of selected algorithms.

	Computation efficiency	Modular characteristics		Visual complexity	
	Computation time	Connectivity F-measure	Relative edge length	Ratio of edge-edge crossings	Ratio of node-edge crossings
**H-SA**	3	6	6	6	6
**H-KK**	4	5	5	5	5
**H-FR**	1	4	2	3	1
**H-GA**	5	3	3	4	4
**GBL**	6	2	4	2	2
**GML**	2	1	1	1	3

As the cells marked in gray in Table 3 show, GML and H-FR achieve relatively good performances on the whole. GML has the best modular characteristics (highest connectivity F-measure and lowest relative edge length) over the other five algorithms, which is not surprising because GML groups densely connected nodes as modules with a clustering algorithm and optimizes the module locations through the special method of module-layout. In addition, GML also achieves the lowest ratio of edge-edge crossings. The high connectivity within modules and low connectivity between modules that result from the GML layout are the main causes of the success of GML in this direction.

H-FR is another outstanding grid layout algorithm, with the lowest ratio of node-edge crossings and the best computation efficiency. However, it is not suitable for the drawing of module networks because of its relatively low connectivity F-measure.

Overall, GML outperforms other algorithms in modular characteristics and achieves relatively outstanding performance in visual complexity (best ratio of edge-edge crossings) and computation efficiency (second-order fast algorithm) for all example networks, which makes GML the best algorithm for the visualization of biological networks with module structures.

### Layout results

**Fig 7:**
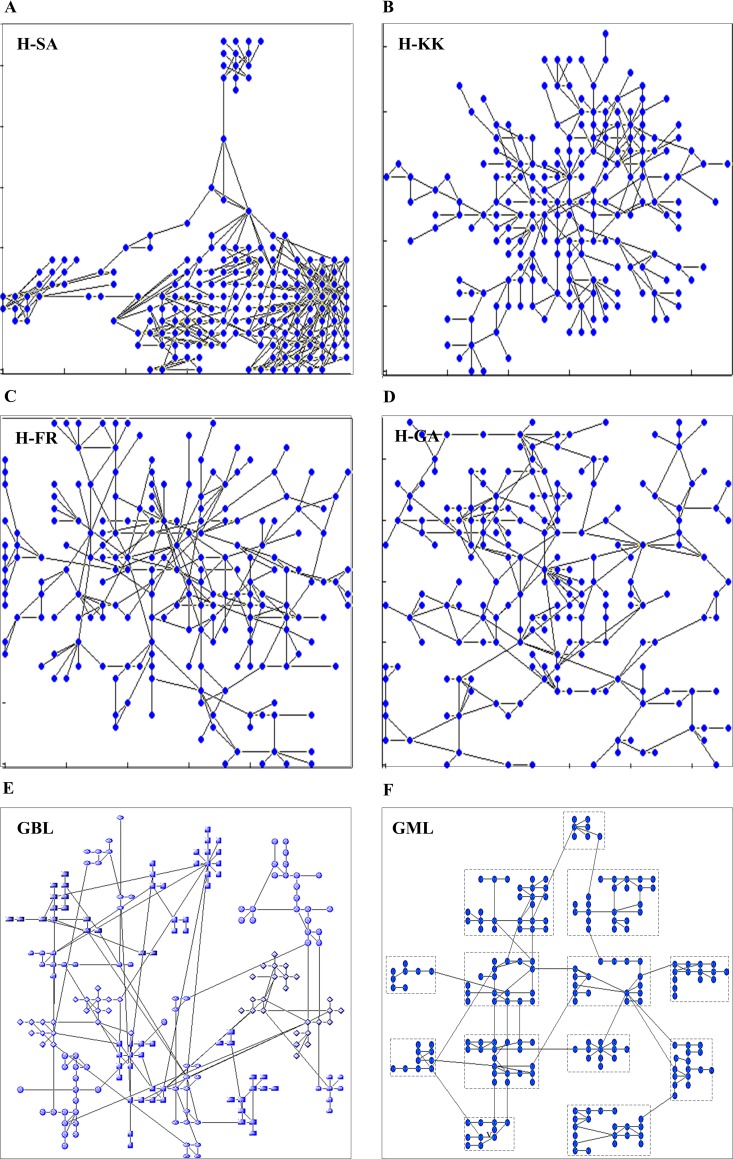
The layout results of hybrid layout, GBL and GML algorithms. The nodes and edges of the network are drawn as blue points and gray straight lines, respectively, in the output layouts.

As shown in Fig 7, a biological network (yeast cell cycle regulatory network) is drawn by the hybrid layout, GBL and GML algorithms for comparison purpose.

In the graphs drawn by the H-KK, H-FR, and H-GA algorithms, the nodes are relatively uniformly distributed in the 2D areas and the modular structures are not apparent. With the help of clustering algorithms, the node distributions by H-SA, GBL and GML show relatively distinct modular features. Moreover, the modules in GML are placed neatly on the plane with lower edge-edge crossings owing to application of the method of module-layout.

## Discussion and conclusions

GML is a novel layout algorithm that aims to clarify the network complexity according to its inherent modular structure. With the method of module layout, GML optimizes the locations of modules to effectively reduce visual complications and is particularly suited for visualizations of complex biological networks with modular features. Working with or without predefined modules, GML provides great flexibility for the advanced analysis of complicated biological networks.

Compared to non-grid layouts (e.g., force-directed), the grid-optimization series including the GML algorithm outperform in the directions of modular characteristics and visual complexity but have relatively high time costs [17–18,20]. Therefore, they are not suited to draw large-scale networks in real-time environments.

The algorithms of grid-optimization series are heuristic algorithms composed of several heuristic procedures. The layout results are usually acceptable solutions but not global optimization solutions. The grid layout is still selected as the basic algorithm in our research because of its advantages in generating compact layouts without node overlapping. In particular, it can be used to generate neat module layouts (see Fig 7), which is important and crucial to the visualization of biological networks.

In the first stage of GML, the multi-level algorithm is selected as module detection algorithm to give rise to predefined modules. Recently, the researches on the module detection algorithms have achieved great progress. He DX *et al*. have presented a novel approach named as NetMRF (network-specific Markov Random Field) that can encode the structural properties of an irregular network in a cost function and generate the best community structures by optimizing the function [40]. To detect link communities and effectively extract community summaries in sentences for topic labeling, Jin D *et al*. have proposed a new unified probabilistic model that explored the intrinsic correlation between communities and topics [41]. What's more, to solve the problem of semi-supervised community detection in attributed networks, Jin D *et al*. have integrated the methods of MRF (Markov Random Fields) and GCN (Graph Convolutional Networks) and designed an end-to-end deep learning algorithm termed MRFasGCN [42]. Among above methods, the NetMRF can be integrated into GML directly as the algorithm of module detection and we would like to design an optimized grid layout algorithm to visualize larger networks based on NetMRF in future.

Overall, we present a grid- and modularity-based layout algorithm, GML, to meet the needs of visualization of complex biological networks. The algorithm outperforms other grid-optimization algorithms in modular characteristics with the special design of the module-layout procedure. With the VisANT plugin of GML, researchers can gain insights into global characteristics as well as discern network details of biological networks.

## Supporting information

S1 Table Data of test results for ten networks for the Hybrid Grid Layout, GBL and GML algorithms. (PDF)

S1 Fig The layout results of the hybrid layout, GBL and GML algorithms for networks of different size. (PDF)
