# Supplementary material for: A new grid- and modularity-based layout algorithm for complex biological networks
Source: PLoS One. 2019 Aug 29;14(8):e0221620. doi: 10.1371/journal.pone.0221620 (PMC6715240; doi:10.1371/journal.pone.0221620)
Supplement: S1 Table — (PDF) [file pone.0221620.s001.pdf]

Data of test results for 10 networks by Hybrid Grid Layout, GBLand GML algorithm

As shown in Table 1 in the manuscript, ten networks are tested by Hybrid Grid Layout (The software is tested with its default parameters and *Division type*= *quad-tree*)[1]. GBL and GML algorithm. GML and GBL [2] are developed by Sheng He and can downloaded from websites: https://hscz.github.io/Biological-Network-Visualization/

All evaluations are based on averages of 10 runs on a Dell laptop (OS: windows 7 64bit, CPU: Intel Core i3-2330M 2.20GHz, Memory: 8.00GB).

ne: ratio of node-edge crossings; ee: ratio of edge-edge crossings
rel: relative edge lengths; cf: connectivity F-measures

1) regulatory network of yeast cell cycle : nodes number:200; edges number:270

	Hybrid Grid Layout				GBL	GML
	H-SA	H-KK	H-FR	H-GA		
ee	0.071206	0.004573	0.009899	0.003920	0.004864	0.002202
ne	0.031725	0.005930	0.006884	0.005967	0.007251	0.004956
rel	0.141084	0.078935	0.106527	0.087862	0.087303	0.062846
cf	0.239692	0.466252	0.457504	0.671113	0.664569	0.641812
time(s)	0.680980	1.717954	0.216498	0.899509	0.344256	0.201846

2) protein-protein interaction network of Utez-screen : nodes number:263; edges number:292

	Hybrid Grid Layout				GBL	GML
	H-SA	H-KK	H-FR	H-GA		
ee	0.064087	0.001683	0.004354	0.001422	0.003554	0.0011768
ne	0.037155	0.004540	0.005713	0.004172	0.004986	0.0038576
rel	0.166658	0.067737	0.096306	0.070419	0.084947	0.0474753
cf	0.161126	0.463286	0.442319	0.650418	0.619581	0.6462536
time(s)	2.583676	2.818806	0.134371	2.681641	0.503746	0.3596354

3) metabolic network of subnetworks in PAO1: nodes number:290; edges number:374

	Hybrid Grid Layout				GBL	GML
	H-SA	H-KK	H-FR	H-GA		
ee	0.025391	0.003317	0.011359	0.003675	0.006007	0.001648
ne	0.002104	0.000549	0.000782	0.000326	0.005375	0.002878
rel	0.109141	0.063959	0.101378	0.073381	0.097195	0.053478
cf	0.260345	0.484512	0.390710	0.669248	0.683672	0.625974
time(s)	1.601790	2.814867	0.199977	0.753544	0.878464	0.477824

4) protein-protein interaction network of Ito-core: nodes number:426; edges number:568

	Hybrid Grid Layout				GBL	GML
	H-SA	H-KK	H-FR	H-GA		
ee	0.117990	0.008661	0.007324	0.011588	0.006564	0.004129
ne	0.035305	0.008293	0.006496	0.010344	0.006537	0.006020
rel	0.199146	0.080619	0.086213	0.090727	0.086587	0.048426
cf	0.114318	0.238298	0.328568	0.474964	0.518934	0.562553
time(s)	4.209931	12.954428	0.492066	4.051982	2.003677	1.016675

5) protein-protein interaction of Y2H-CCSB: nodes number:964; edges number:1598

	Hybrid Grid Layout				GBL	GML
	H-SA	H-KK	H-FR	H-GA		
ee	0.041328	0.040944	0.029364	0.041328	0.01179	0.0098032
ne	0.010942	0.010657	0.007147	0.010942	0.00574	0.007204
rel	0.095765	0.093199	0.081049	0.095765	0.091731	0.0644233
cf	0.322472	0.104722	0.180142	0.322472	0.454424	0.4481533
time(s)	17.748726	5.021231	1.735318	17.94386	17.04353	9.3616455

6) metabolic network of PAO1: nodes number:1294; edges number:1590

	Hybrid Grid Layout				GBL	GML
	H-SA	H-KK	H-FR	H-GA		
ee	0.131682	0.008240	0.004628	0.004009	0.004037	0.0013085
ne	0.029061	0.005733	0.002763	0.002926	0.002844	0.001523
rel	0.175201	0.061800	0.053562	0.049016	0.073965	0.039755
cf	0.113848	0.206919	0.302924	0.508739	0.615155	0.591437
time(s)	15.578077	51.033321	2.088177	13.18467	26.42677	15.97377

7) metabolic network of *L.lactis*: nodes number:1489; edges number:3172

	Hybrid Grid Layout				GBL	GML
	H-SA	H-KK	H-FR	H-GA		
ee	0.521300	0.498710	0.078449	0.116134	0.022499	0.020611
ne	0.000863	0.000894	0.000477	0.000513	0.007896	0.009714
rel	0.216019	0.228096	0.083711	0.117968	0.099536	0.093414
cf	0.101479	0.038875	0.178328	0.236352	0.488896	0.493255
time(s)	12.466079	97.942734	5.325380	39.421353	39.7722	21.1165

8) metabolic network of *S.cerevisiae* iFF708: nodes number:2879; edges number:5616

	Hybrid Grid Layout				GBL	GML
	H-SA	H-KK	H-FR	H-GA		
ee	0.204736	0.074734	0.034704	0.064695	0.014592	0.014723
ne	0.017623	0.008207	0.004508	0.007463	0.00360	0.007398
rel	0.182013	0.093065	0.067448	0.094054	0.090183	0.054825
cf	0.057766	0.057538	0.126936	0.222493	0.42789	0.436074
time(s)	109.736182	124.8066	19.340113	152.3665	164.804	102.3894

9) metabolic network of *Aspergillus nige*r: nodes number:3774; edges number:7976

	Hybrid Grid Layout				GBL	GML
	H-SA	H-KK	H-FR	H-GA		
ee	0.241738	0.070276	0.034795	0.071025	0.020055	0.015031
ne	0.013281	0.005430	0.003346	0.007060	0.004312	0.006316
rel	0.171269	0.079494	0.059594	0.091491	0.086835	0.065755
cf	0.049443	0.057034	0.125687	0.224962	0.372337	0.418164
time(s)	172.8246	160.8246	36.12983	245.3156	247.8322	150.8463

10) metabolic network of *Aspergillus oryzae*: nodes number:4976; edges number:11042

	Hybrid Grid Layout				GBL	GML
	H-SA	H-KK	H-FR	H-GA		
ee	0.862952	0.100147	0.051377	0.105103	0.015077	0.016834
ne	0.032401	0.005909	0.003909	0.006619	0.004201	0.005384
rel	0.298693	0.085692	0.062177	0.092344	0.102764	0.055204
cf	0.02294	0.043599	0.100890	0.186416	0.345884	0.366774
time(s)	247.6241	265.0459	76.91207	424.92874	470.3824	230.6217

References

1. Inoue K, Shimozono S, Yoshida H, Kurata H: **Application of Approximate Pattern Matching in Two Dimensional Spaces to Grid Layout for Biochemical Network Maps**. *PLoS ONE* 2012, **7**(6):e37739.
2. He S, Liu Y, FeiyueYe, Guo D: **Research on complex network layout algorithm based on grid point matching method**. *Journal of Complex Networks* 2018(6):145-154.
