# Supplementary material for: A new grid- and modularity-based layout algorithm for complex biological networks
Source: PLoS One. 2019 Aug 29;14(8):e0221620. doi: 10.1371/journal.pone.0221620 (PMC6715240; doi:10.1371/journal.pone.0221620)

1. Layout results of ***Subnetwork of PAO1***(Metabolic Network: nodes: 290, edges :374)

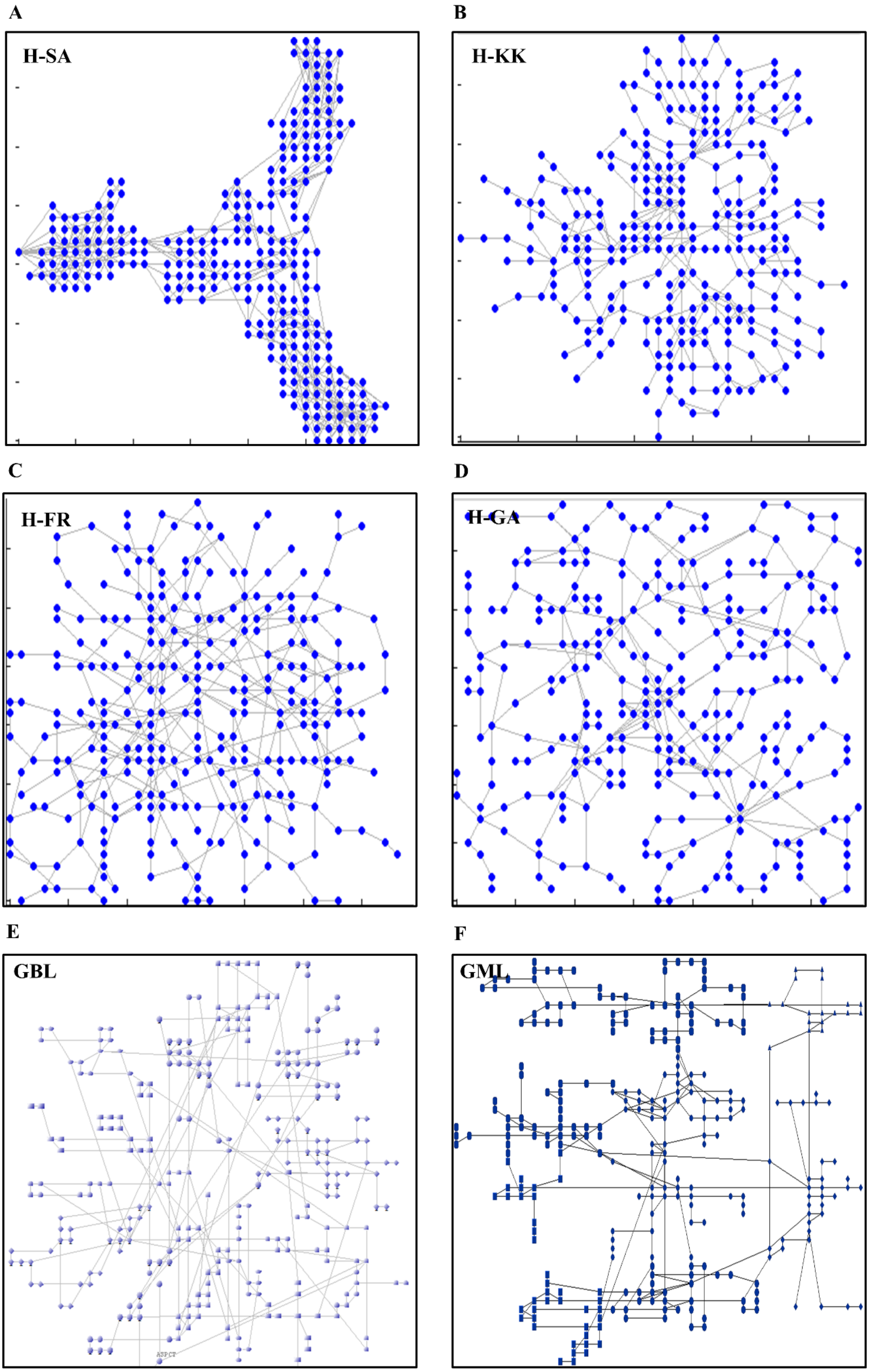

2. Layout results of ***Network of Y2H-CCSB*** (protein-protein interaction: nodes: 964, edges :1598)

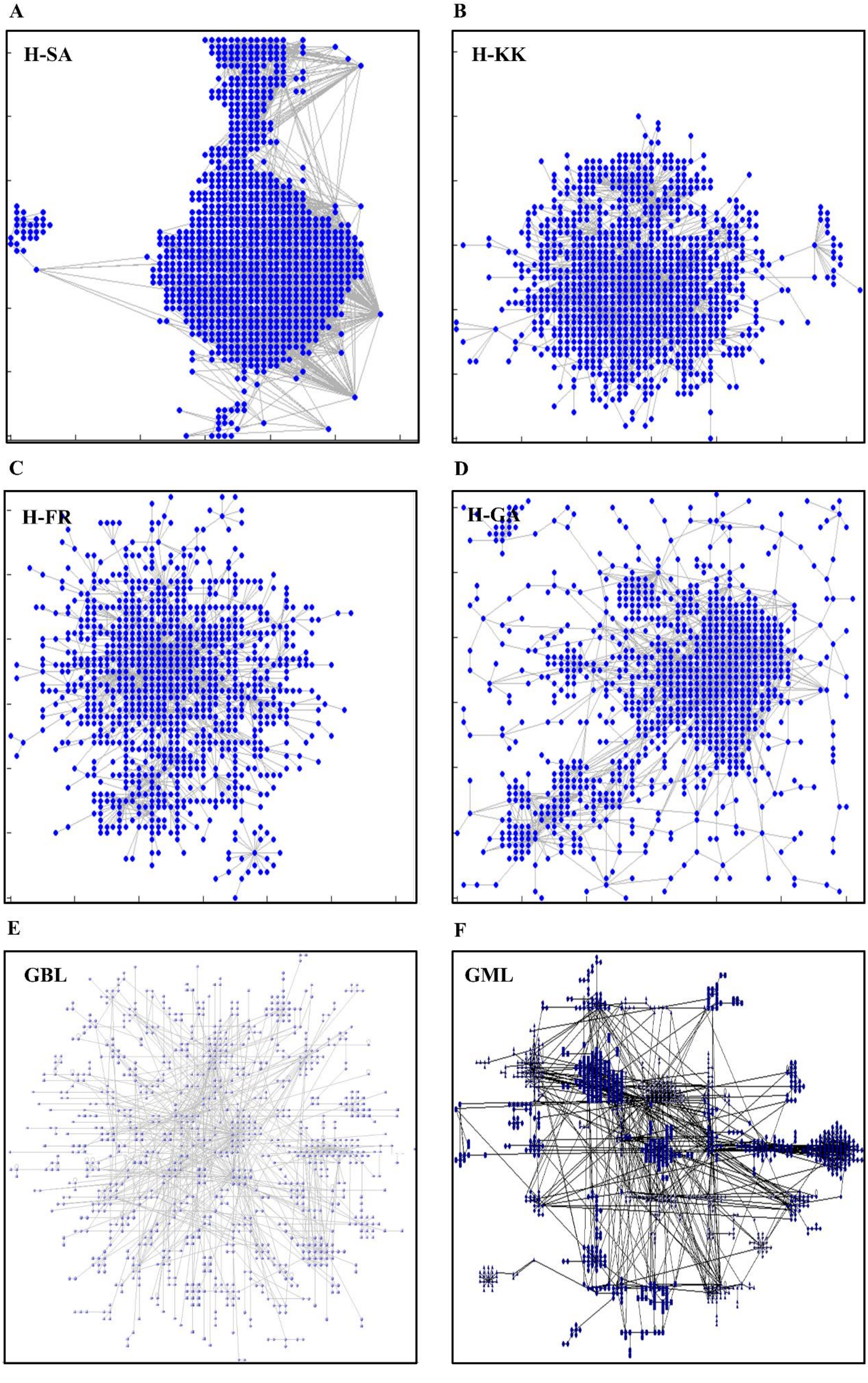

3. Layout results of ***Network of L.lactis*** (Metabolic Network: nodes: 1489, edges :3172)

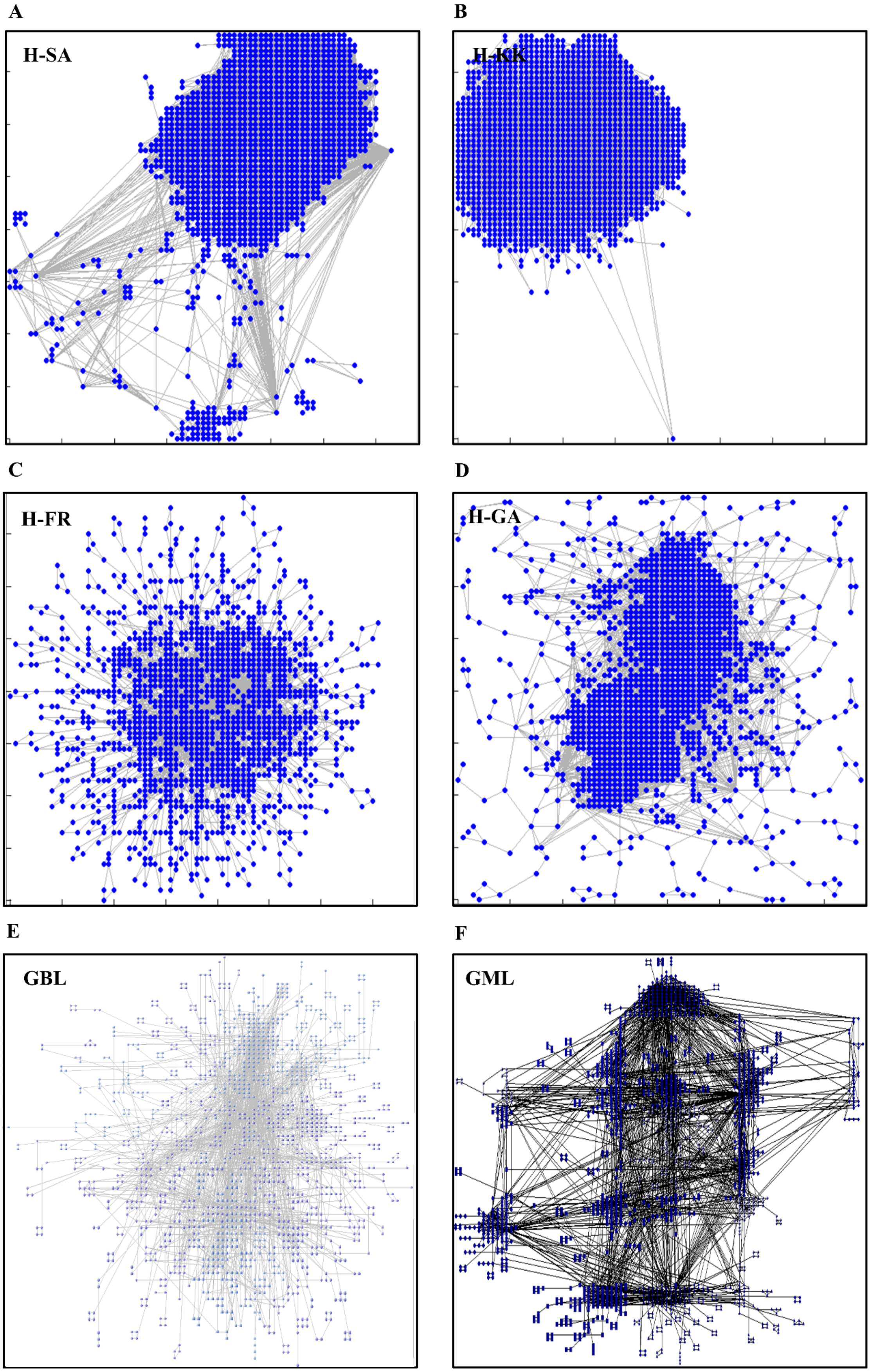

4. Layout results of ***Network of S.cerevisiae iFF708*** (Metabolic Network: nodes: 2879, edges :5616)

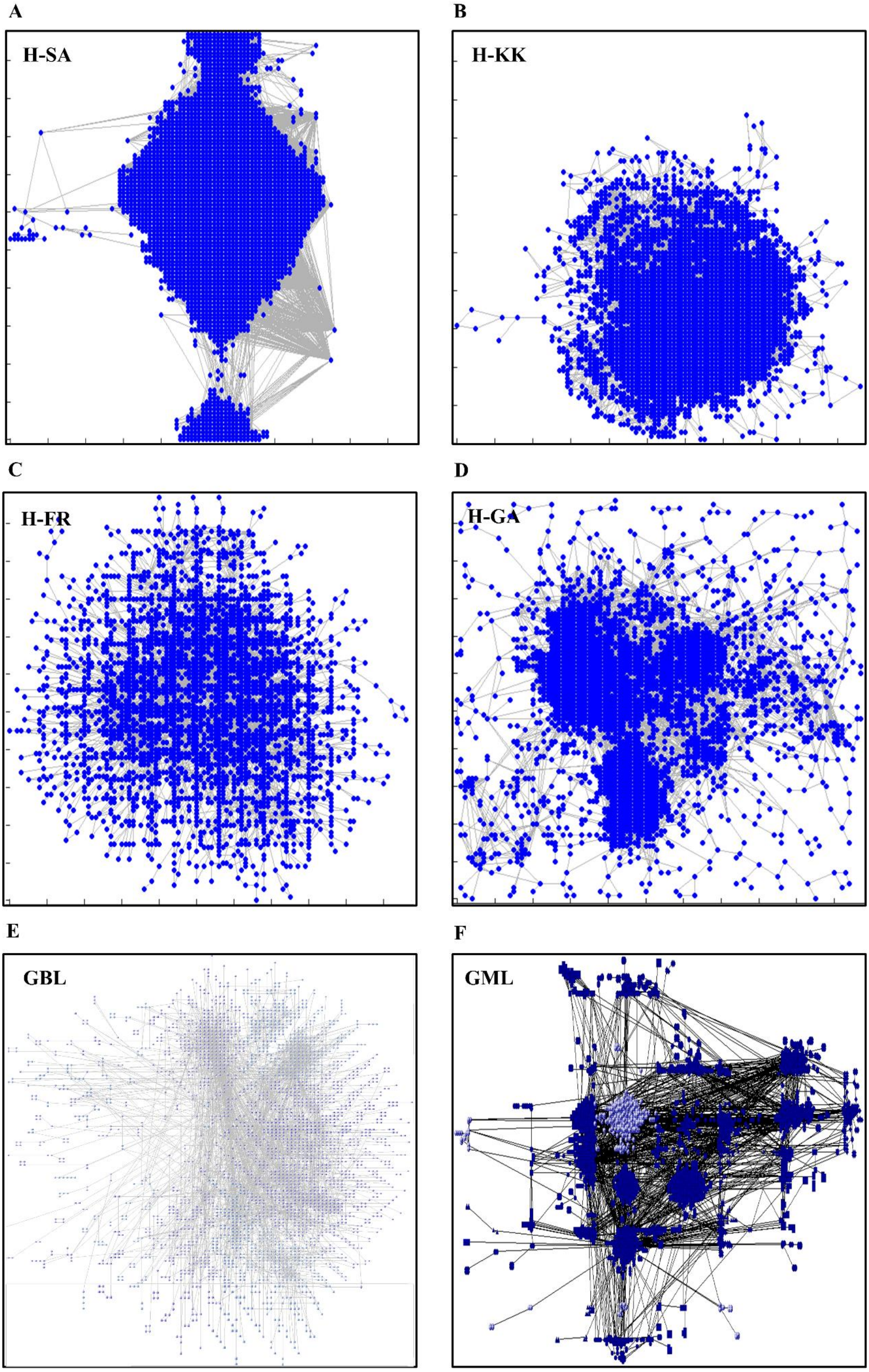

Supplement: S1 Fig — (PDF) [file pone.0221620.s002.pdf]
